# Uranium and other contaminants in hair from the parents of children with congenital anomalies in Fallujah, Iraq

**DOI:** 10.1186/1752-1505-5-15

**Published:** 2011-09-02

**Authors:** Samira Alaani, Muhammed Tafash, Christopher Busby, Malak Hamdan, Eleonore Blaurock-Busch

**Affiliations:** 1 Fallujah General Hospital, Althubbadh, Fallujah, 00964, Iraq; 2 Department of Molecular Biosciences, University of Ulster, Cromore Rd, Coleraine, BT52 1SA, UK; 3 The Cancer and Birth Defects Foundation, Office 4, 219 Kensington High Street, London, W8 6DB, UK; 4 Laboratory for Clinical and Environmental Analysis, Microtrace Minerals, Rohrenstrasse 20, D-91217, Hersbruck, Germany

**Keywords:** Fallujah, Iraq, congenital anomaly, cancer, heavy metals, Depleted Uranium, hair analysis

## Abstract

**Background:**

Recent reports have drawn attention to increases in congenital birth anomalies and cancer in Fallujah Iraq blamed on teratogenic, genetic and genomic stress thought to result from depleted Uranium contamination following the battles in the town in 2004. Contamination of the parents of the children and of the environment by Uranium and other elements was investigated using Inductively Coupled Plasma Mass Spectrometry. Hair samples from 25 fathers and mothers of children diagnosed with congenital anomalies were analysed for Uranium and 51 other elements. Mean ages of the parents was: fathers 29.6 (SD 6.2); mothers: 27.3 (SD 6.8). For a sub-group of 6 women, long locks of hair were analysed for Uranium along the length of the hair to obtain information about historic exposures. Samples of soil and water were also analysed and Uranium isotope ratios determined.

**Results:**

Levels of Ca, Mg, Co, Fe, Mn, V, Zn, Sr, Al, Ba, Bi, Ga, Pb, Hg, Pd and U (for mothers only) were significantly higher than published mean levels in an uncontaminated population in Sweden. In high excess were Ca, Mg, Sr, Al, Bi and Hg. Of these only Hg can be considered as a possible cause of congenital anomaly. Mean levels for Uranium were 0.16 ppm (SD: 0.11) range 0.02 to 0.4, higher in mothers (0.18 ppm SD 0.09) than fathers (0.11 ppm; SD 0.13). The highly unusual non-normal Fallujah distribution mean was significantly higher than literature results for a control population Southern Israel (0.062 ppm) and a non-parametric test (Mann Whitney-Wilcoxon) gave p = 0.016 for this comparison of the distribution. Mean levels in Fallujah were also much higher than the mean of measurements reported from Japan, Brazil, Sweden and Slovenia (0.04 ppm SD 0.02). Soil samples show low concentrations with a mean of 0.76 ppm (SD 0.42) and range 0.1-1.5 ppm; (N = 18). However it may be consistent with levels in drinking water (2.28 μgL^-1^) which had similar levels to water from wells (2.72 μgL^-1^) and the river Euphrates (2.24 μgL^-1^). In a separate study of a sub group of mothers with long hair to investigate historic Uranium excretion the results suggested that levels were much higher in the past. Uranium traces detected in the soil samples and the hair showed slightly enriched isotopic signatures for hair U238/U235 = (135.16 SD 1.45) compared with the natural ratio of 137.88. Soil sample Uranium isotope ratios were determined after extraction and concentration of the Uranium by ion exchange. Results showed statistically significant presence of enriched Uranium with a mean of 129 with SD5.9 (for this determination, the natural Uranium 95% CI was 132.1 < Ratio < 144.1).

**Conclusions:**

Whilst caution must be exercised about ruling out other possibilities, because none of the elements found in excess are reported to cause congenital diseases and cancer except Uranium, these findings suggest the enriched Uranium exposure is either a primary cause or related to the cause of the congenital anomaly and cancer increases. Questions are thus raised about the characteristics and composition of weapons now being deployed in modern battlefields

## Background

### Health effects from wars in Iraq

There have been reports of increased rates of cancer and congenital anomaly (CA) from Fallujah, Iraq [1,2]. This spectrum of health conditions points to the existence of some historic exposure which has caused significant teratogenic, genetic or genomic stress to the population. In addition to the increased cancer and rates and infant deaths, the epidemiological study [1] showed that there was a sudden significant drop in the sex ratio (an indicator of genetic stress) in the cohort born in 2005, one year after the battles which occurred in the city, suggesting that the cause of all these effects is related to the time of the US led invasion of the city in 2004. Because of the lack of knowledge of the respondents in this questionnaire about the precise cause of death of their children, that study focused on infant mortality as an indicator of birth defects. Results showed levels of infant mortality of around 80 per 1000 live births in children, which can be compared with a figure of 17 in Jordan and 9 in Kuwait. However, it seems that the findings in Fallujah may only reflect general deterioration in birth outcomes and child health in Iraq. The Iraqi child and maternal mortality survey [3] covered 46,956 births in Iraq from 1994-1999. Results were obtained by questionnaires filled out by the mothers and results were given for all children aged 0-4 who died in 1994-1999. Effects found in this period, if due to environmental agents, would, of course, follow exposures in and following the first Gulf War, GW1. Using data presented in the tables in this publication it is easy to show that the results indicated a marked increase in deaths in the first year of life with an infant mortality (0-1) rate of 93 per 1000 live births. 56% of deaths in all the children aged 0-5 occurred in the first month after birth but since the results were from self reporting, it was difficult to draw conclusions as to the underlying causes of death except in the case of oncology/haematology. For example, the largest reported proportion of deaths in the neonates were listed as "cough/difficulty breathing" which might result from many different underlying causes. The low rates from congenital malformation reported are hardly credible (Table 1). However, using data published in the report [3] it appeared that the cancer and leukemia death rates in the entire all-Iraq 0-4 group were about three or four times the levels found in western populations for this age group (Table 1). These rates were three times higher in the South where Depleted Uranium (DU) was employed in the major tank battles near the Kuwait border (53 per 100,000 per year) than in the North (18 per 100,000 per year) where there was less fighting and where DU was not employed to such an extent [4]. Furthermore, cancer and leukemia rates were highest in the 0-1 year group, which is unusual; the main peak in childhood cancer is generally found at age 4.

**Table 1:** Data on child mortality, congenital anomaly and oncology/haematology as causes of death reported by mothers in the Iraqi Child and Maternal Mortality Survey 1994-1999 [3]

*Region*	*Live births*	*Rate/1000 live births*	*CA rate* */1000 live births*	*Oncology/haematology* *Mean Death Rate per 100,000 per year *
North	12159	101	3.9	18.0
Middle	18637	126	7.6	20.4
Mid Euphrates	8224	137	12.6	24.4
South	7936	144	11.3	52.9

**All**	**46956**	**125**	**6.9**	**26.0**

As far as Fallujah is concerned, we have ourselves made a study [Alaani S.; Busby C; Hamdan M; Al-Fallouji M: *Infant mortality, sex ratio, congenital anomaly and environmental contamination in Fallujah, Iraq*, submitted] of the levels of different types of congenital anomalies diagnosed by one pediatrician in an 11 month period in the Fallujah General Hospital. Results, confirm the existence of high rates of congenital anomaly in this birth cohort.

### Anomalous health effects of Uranium weapons

Since the use of Depleted Uranium in GW1, there has been a research focus on contamination by this material as a potential cause of increases in congenital anomaly (CA) and cancer rates [5]. When Depleted Uranium weapons are employed, sub-micron aerosolized particles of ceramic Uranium oxides are created [6,7]. These are respirable and the inhalation of Uranium involves a 200-fold increased radiation dose conversion coefficient (the committed effective radiation dose per unit intake) compared with ingested Uranium. This is due to the long biological half life of internal Uranium and the very low gut transfer factor for ingested Uranium [6-8]. Of course, the troops were also exposed to DU aerosols. A number of studies of the GW 1 veterans have shown statistically significantly increased rates of congenital malformation in their children [9-11]. For example, Doyle et al. [9] reported rates of congenital malformations in a group of 13,191 offspring of male and 360 offspring of female UK Gulf war veterans, finding relative risks of 1.5 (95% CI 1.3-1.7) for all CA's. Araneta et al. [10] reported significant excess congenital heart defect and hypospadia rates in 11,961 US Gulf war veteran live-born offspring compared with military controls. Relative risks were between 2.7 (1.1-6.6) for tricuspid valve insufficiency and 6.0 (1.2-31.0) for aortic valve stenosis. Kang et al. [11] compared 3371 US Gulf War veteran offspring with 3625 non Gulf War veterans and reported higher prevalence of moderate to severe birth defects RR1.78 (1.19-2.66) with father as veteran and RR 2.8 (1.26-6.25) with mothers as veterans. Other studies have found similar results but there have also been studies which do not find any increased risks although many of these latter studies suffer from problems with small numbers [9]. An interesting and relevant study is that of significant excess rates of cancer and congenital birth anomalies in the Quirra polygon in Sicily where NATO is believed by the authors of the study to test Uranium weapons [12]. Nevertheless, besides DU, there may also be a number of other potential causes for any increased risks of congenital anomaly in Fallujah or in Southern Iraq. Modern warfare involves the deployment of novel weapons systems which create contamination of the environment and the local inhabitants of the war zones by a range of heavy metals and other substances. For example, analysis of wound tissue of war injuries produced in Gaza in 2009 revealed traces of elements which have been argued by the authors to be associated with carcinogenicity and fetotoxicity, including As, Cd, Sn, Pb, Hg, U, Cr, Ni, Co and V [13]. To investigate this issue we examine the concentrations and isotopic ratios U238/U235 of Uranium and other elements in the hair of the parents of the CA children. To provide information about environmental levels of Uranium we also measured the Uranium content and U-238/U235 isotopic ratio of surface soil samples and content of tap water, well water and water from the River Euphrates which flows though the city.

It is not our intention here to exhaustively discuss the arguments relating to the genotoxicity and fetotoxicity of Uranium; these have been rehearsed at some length in the literature. However, since Uranium is the only known radioactive heavy metal exposure in Iraq, it must be considered to be a major suspect for the cause of the effects found in Fallujah and also in the rest of Iraq. Such a suspicion is also supported by other evidence of birth defects in Gulf War Veterans and increasingly from studies of the genotoxicity of Uranium in cell and animal studies. Induction of genetic and genomic damage by ionizing radiation has been recognized since the early work of Muller on the genetic effects of X-rays on *Drosophila *[14,15]. Increased levels of genetic and genomic based conditions have been reported in those exposed to internal contamination from fallout from the Chernobyl accident [16-19]. It should be noted that the radiation doses in these Chernobyl affected territories were not considered high [20] and were generally less than natural background. However the concept of absorbed dose itself may not be valid for the internal exposures from the Chernobyl fallout [21-23]. Indeed, significant effects of the Chernobyl fallout were reported for infant leukemia rates in those children who were *in utero *during the fallout period reported by 5 different research groups for Greece, Germany, Scotland, Wales and Belarus; taken together and because the absorbed doses were so low, these raise questions about the application of the concept of dose used for risks from external exposures in the A-Bomb lifespan studies for internal substances like Strontium-90 and Uranium which have high affinity for DNA [24,25]. For example, the fetotoxic effects of internal exposures to Strontium-90, a radionuclide that binds to DNA, were investigated in the period of atmospheric testing [26]. In mice, Sr-90 exposure of fathers caused high rates of fetal death whereas the same dose from the radionuclide Cs-137 which does not bind to DNA produced no effect. Uranium, as its molecular species in tissue, the UO_2_^++ ^ion, also binds strongly to DNA having an affinity constant of 10^10 ^M^-1 ^[27] a fact which has been known since the 1960s when it was first used as an electron microscope stain for chromosomes [28]. For this and a number of other reasons Uranium may be considered theoretically to show enhanced levels of genomic damage relative to that which its absorbed dose might predict [29]. Such anomalous genomic damage has, indeed been found at low concentrations in cell cultures [30-32] rodents [33,34] in Uranium miners [35] and Gulf War veterans [36]. Given this evidence for the genomic effects of Uranium, since the respirable aerosol form of DU is far more effective in becoming internalized and the particles may remain in the body for more than 10 years [6] it is not surprising that there is considerable evidence emerging that the effects of exposure to DU, or aerosolized Uranium weapons involve a wide range of adverse human health conditions [37,38].

These considerations provide a general background for our examination of Uranium levels in the mothers and fathers of the children with CA in Fallujah. In order to attempt to characterize the source of any Uranium we also determined the U238/U235 isotopic ratio which for natural Uranium is always exactly 137.88. Pure Depleted Uranium in anti-tank ammunition may be considered to have an isotopic ratio of over 400 but mixtures with natural environmental Uranium will of course show lower ratios [6,38].

### Hair analysis

With advances in technology in the last 10 years, analysis of hair contamination has become a valuable tool in assessing environmental exposures, particularly for Uranium [39-43]. We determined Uranium and other elements the hair of mothers and fathers of children who had been born with major congenital anomalies.

In principle, contamination in hair represents excretion into the hair strand at the time of exposure; thus it might be expected that analysing hair along the lock from scalp to distal end, might reveal information about changes in exposure with time. It is of value in this regard that the Moslem women in the group all had long hair, 30-80 cms in length. Since hair grows on average, 1 cm per month, this means that the distal end of the hair of an 80 cm lock taken in February 2011 began growing in 2005. For this reason and also to investigate the accuracy of the initial determinations we obtained a second sample of hair about 9 months after the initial sample from a group of women whose analysis had shown the presence of Uranium in the first analysis. In this second investigation, we divided the hair locks into 12-14 cm sections, each of which was separately analysed from its mid point.

## Subjects and methods

### Hair analysis; initial study

Parents of children born in 2009-2010 with major congenital anomalies in Fallujah General Hospital volunteered to take part in the study. Mothers and fathers separately gave hair samples in May 2010 and also completed a questionnaire. Details from the questionnaire were filed with the clinical details of the child's congenital anomaly. In two cases, hair from the child was also obtained. We obtained the clinical details of the congenital anomaly, the age of the parents, their smoking history and alcohol drinking history and where they had lived. All of the parents were from Fallujah and had been present at the time of and after the attacks in 2004. Hair was cut from the nape of the neck with stainless steel scissors and placed in a sealed polythene bag. The centre of the first 15 cm of the scalp end of the lock was employed in the case of the mother. Fathers' hair as received was generally very short, less than 2 cm. There were 25 samples from parents and two samples from children. In all cases, samples were physically brought to the UK where they were opened in the laboratory, the samples divided in two longitudinally and one half placed in a second polythene bag and re-coded. The re-coded half-sample was then posted to Germany for analysis. There, samples were removed from the plastic bags, washed well with a de-ionized aqueous detergent solution (Triton X-100) and dried before cutting and weighing. In the initial study, for about half the cases, and where there were only small quantities of sample, mother and fathers hair were mixed together. This was because the quantity of hair available from some of the fathers was very small and because it was believed it would give gave a more representative value for the population with fewer determinations. Each determination was based on about 100 mg of hair precisely weighed. Samples were dissolved in 3.0 ml A/R Nitric acid containing 10% of 30% hydrogen peroxide solution by microwave digestion at 80 degrees for 1 hour to give a clear solution. The solutions were diluted to 10 ml with ultra-pure water and examined by Inductively Coupled Plasma Mass Spectrometry ICPMS (Agilent 7700 with Octupole Reaction System).

### Hair analysis; long hair study

In the case of the long hair study, samples were obtained in February 2011 from 4 women whose hair had already been analysed in the first study plus two other women (not with children with birth defects) who lived in Fallujah and who volunteered to take part. The hair was divided longitudinally and cut into equal sections of 13 to 15 cm (depending upon the initial length) and labeled from the scalp end. The re-coded half-sample was then posted to Germany for analysis using the same procedure already outlined.

### Drinking and local water samples

Tap water, well water and water from the river Euphrates in the city were also analysed in Germany by ICPMS after treatment with nitric acid/hydrogen peroxide and filtration.

### Soil analysis

For soil samples, the analysis was carried out by the Harwell Scientifics Laboratory in Oxford, UK. Six samples of surface soil from the top 1 cm were obtained from various representative areas in that part of Fallujah where there had been bombardment and fighting in 2004. Gamma dose rates at each site were checked at 30 cm from the ground with a calibrated Russian SOSNA twin chamber Geiger Counter which has a thin window and responds to beta and gamma radiation. Initially, three separate aliquots of each sample were digested in concentrated nitric and hydrofluoric acids in fluoropolymer vessels by microwave. Following digestion, samples were made to a known volume of demineralised water having resistivity of 18.2 MOhm cm. All samples were analysed for total Uranium and Uranium isotopes at the same laboratories using Inductively Coupled Plasma Mass Spectrometry ICPMS (Perkin Elmer Agilent 7500CE) on the above nitric acid/hydrofluoric acid digestions. Calibration was achieved using standard addition of a certified Uranium standard and instrumental drift was corrected with a Bismuth spike. Quality Control standards at 5.0, 10 and 20 micrograms per litre were prepared from an alternative stock source solution to that used to prepare the calibration standard. Three independent preparations were made from each sample and each was run three times on the system. After obtaining initial concentration data, the U-238/U-235 isotope ratio was obtained in a separate determination where an acid dissolution of a larger quantity of material was passed onto an ion exchange column to separate and concentrate Uranium from the matrix. Approximately 3 g of sample was ashed overnight at 450 C, and the residue digested in concentrated nitric and hydrofluoric acids. After co-precipitation of the Uranium with iron hydroxide, ion-exchange chromatography (Eichrom 1 × 8 100-200 mesh) was used to further purify and separate the Uranium. This Uranium extract (which had about 50 times the Uranium channel counts than the initial sample was then further analysed for Uranium isotope U238/U235 ratios.

## Results

### Initial hair study

Results were obtained for all the elements listed in Table 2. The listed range of normal background values (95% CI) for the comparison levels in hair were obtained from an unpublished database created through studies carried out by the present laboratory on 1000 healthy subjects living in Germany and the USA. Also shown are the limits of detection of the element at these laboratories. In addition for further comparison are shown means and ranges taken from Rodushkin and Axelsson [43] which are slightly different for some elements. The mean ages of the fathers was 29.6 (SD 6.2) and the mothers 27.3 (SD 6.8). Only 5 fathers and 3 mothers in were over the age of 30 and only two parents (a father and mother) over 40. None of the parents (all of whom are Moslems) reported drinking alcohol. Four fathers and none of the mothers were smokers. These individuals did not show higher than average amounts of Uranium than the non smokers. In Table 3 are shown results for individual parents for elements of interest which are found at levels which are higher than the normal range, together with details of the child anomaly. Mean levels for Uranium were 0.16 ppm (SD: 0.11) range 0.02 to 0.4, higher in mothers (0.18 ppm SD 0.09) than fathers (0.11 ppm; SD 0.13). It was also of interest to examine the variation in concentration of these elements between the mothers and fathers. Table 4 gives details of some comparisons of mothers and fathers of the same children.

**Table 2:** Limits of Detection (LOD) Mean and Standard Deviations of concentrations mg/kg of elements measured in the cohort of 26 parents of children from Fallujah with congenital anomalies

*Element*	*LOD*	*Normal lab range*	*Literature^43^* *Means and SDs* *unexposed*	*Mean concentration* *In CA parents*	*Standard Deviation*
Calcium*	0.04	220-1600	750 (660)	3622	2736
Magnesium*	0.015	20-130	46 (38)	364.9	271
Chromium	0.003	0.03-0.68	0.167 (0.118)	0.16	0.14
Cobalt*	0.001	0.02-0.57	0.013 (0.011)	0.08	0.09
Copper	0.004	10.0-41.0	25 (21)	24.38	9.18
Iodine	0.002	0.05-5.0	0.68 (0.58)	0.16	0.14
Iron*	0.039	4.6-17.7	9.6 (4.4)	25.15	18.56
Manganese*	0.003	0.12-1.30	0.56 (0.55)	2.18	2.23
Molybdenum	0.001	0.02-1.00	0.042 (0.02)	0.07	0.04
Selenium	0.029	0.21-5.46	0.830 (0.280)	1.42	2.59
Vanadium*	0.001	0.01-0.73	0.027 (0.024)	0.37	0.26
Zinc*	0.012	150-272	142 (29)	340	230
Boron	0.104	0.07-0.9	0.670 (0.620)	1.1	0.84
Germanium	0.003	< 1.65	0.0046 (0.0031)	0.01	0.01
Lithium	0.001	< 0.53	0.017(0.013)	0.01	0.01
Strontium*	0.000	0.65-6.90	1.20 (1.00)	26.9	2.78
Tungsten	0.000	< 0.06	0.0053 (0.0049)	0.01	0.01
Aluminium*	0.217	< 8.00	8.2 (4.8)	16.25	12.27
Antimony	0.001	< 0.6	0.022 (0.017)	0.04	0.03
Arsenic	0.005	< 1.00	0.085(0.054)	0.1	0.07
Barium*	0.000	< 4.64	0.640 (0.490)	5.42	42.8
Beryllium	0.001	< 0.20	0.0013 (0.0009)	0.00	0.00
Bismuth*	0.000	< 0.27	0.019 (0.025)	3.68	7.07
Cadmium	0.001	< 0.20	0.058 (0.056)	0.08	0.10
Cerium	0.000	< 0.10	0.039 (0.05)	0.02	0.02
Caesium	0.000	< 0.01	0.00067	0.00	0.00
Dysprosium	0.000	< 0.01	-	0.00	0.00
Erbium	0.000	< 0.01	-	0.00	0.00
Europium	0.000	< 0.01	-	0.00	0.00
Gadolinium	0.000	< 0.02	-	0.00	0.00
Gallium*	0.000	< 0.22	.0025 (.0015)	0.01	0.01
Iridium	0.001	< 0.01	-	0.00	0.00
Lead*	0.002	< 3.00	0.960 (0.850)	4.08	4.36
Lutetium	0.000	< 0.01	-	0.00	0.00
Mercury*	0.003	< 0.60	0.261 (0.145)	12.41	42.2
Nickel	0.003	< 1.00	0.430 (0.400)	0.88	0.86
Palladium*	0.003	< 0.04	0.00032 (0.00078)	0.01	0.01
Platinum	0.000	< 0.01	-	0.00	0.00
Praseodymium	0.000	< 0.01	-	0.00	0.00
Rhenium	0.000	< 0.00	-	0.00	0.00
Rhodium	0.000	< 0.01	-	0.00	0.00
Ruthenium	0.001	< 0.45	-	0.00	0.00
Samarium	0.000	< 0.01	-	0.00	0.00
Silver	0.002	< 1.00	0.231 (0.298)	0.11	0.13
Tantalum	0.001	< 0.01	-	0.00	0.00
Tellurium	0.006	< 0.02	-	0.00	0.00
Thallium	0.000	< 0.01	-	0.00	0.00
Thorium	0.000	< 0.03	-	0.00	0.00
Thullium	0.000	< 0.00	-	0.00	0.00
Tin	0.001	< 0.70	0.320 (0.390)	0.67	2.31
Titanium	0.009	< 2.20	0.830 (0.680)	0.53	0.31
Uranium 1^st^	0.001	< 0.15	0.057 (0.065)	0.16	0.11
Uranium 2^nd ^*	0.001	< 0.15	0.057 (0.065)	0.26	0.09
Ytterbium	0.000	< 0.01	-	0.00	0.00
Zirconium	0.001	< 1.47	0.155 (0.237)	0.02	0.09
					

Elements which are found to be more than 2SD from the Literature [43] for Sweden unexposed mean are asterisked.

**Table 3:** Individual cases with concentrations of selected elements of interest, Calcium, Strontium, Bismuth, Mercury and Uranium

*P* *sex*	*Child* *sex*	*Anomaly*	*Ca*	*Sr*	*Bi*	*Hg*	*U*
M	M	Stillbirth, gastroschism	589	2.8	8.2	6.9	0.05
MF	M	Heart defects	9581	45.5	32.4	162.12	0.29
MF	F	Chest defect	4024	28.7	0.89	2.4	0.28
MF	M	Lymphatic abnormality, cystic hygroma	11796	26.9	8.06	4.7	0.3
MF	M	Heart defects	6010	27.7	10.7	144.9	0.12
M	M	Heart defects, brain atrophy, died	2499	22.1	0.16	0.38	0.02
MF	M	Heart defects, tracheo-oesophageal fistula	5381	96.3	2.52	9.12	0.05
MF	F	Heart defects	4557	31.9	0.4	0.31	0.28
MF	F	Heart defects	6757	48.8	1.06	1.86	0.07
M	F	Heart defects, cleft palate	1094	8.89	1.33	0.66	0.40
MF	M	Heart defects	3616	33.3	13.8	22.6	0.20
MF	M	Heart defects, pulmonary stenosis	5856	44.9	2.3	9.3	0.06
F	F	Heart disease, cleft palate	4066	39.4	1.25	2.66	0.16
MF	F	Heart defects	2322	9.9	4.9	41.5	0.07
F	M	Various, cleft lip, omphalocele, died	2904	18.3	0.37	1.2	0.17
F	M	Heart defects	3584	15.5	0.34	0.46	0.24
M	F	Cephocephaly, single nostril, died	663	3.14	2.23	1.75	0.07
F	F	Cephocephaly, single nostril, died	2542	27.4	8.5	5.39	0.23
M	M	Cleft lip, cleft palate, bilateral hand deformity, died	602	4.4	0.32	0.33	0.16
M	M	Heart defects	2306	15.3	0.03	0.1	0.04
F	M	Heart defects, brain atrophy, died	4371	41	16.1	11.9	0.07
F	F	Multiple CA	3480	31.8	0.41	0.4	0.24
F	M	Stillbirth, gastroschism	2611	20.2	5.16	4.67	0.30
M	F	Multiple CA. died	595	4.27	0.26	0.33	0.05
F	F	Multiple CA. died	2068	21.7	4.9	4.12	0.02
							

Parents hair is coded M for father, F for mother and MF for mixed

**Table 4:** Comparisons of pairs of mothers (M) and fathers (F) of the same children for concentrations of selected contaminants in hair

*Child*	*Calcium*	*Strontium*	*Bismuth*	*Mercury*	*Uranium*
1M	2499	22.17	0.16	0.38	0.01
1F	4371	41.1	11.9	16.1	0.07
1C	na	na	na	na	na
2M	1094	8.89	1.33	0.66	0.40
2F	4066	39.4	1.25	2.66	0.16
2C	2847	12.4	1.1	0.94	0.02
3M	663	3.14	2.23	1.75	0.07
3F	2542	27.43	8.50	5.39	0.23
3C	2440	2.78	0.08	0.43	0.00
4M	602	4.36	0.32	0.33	0.16
4F	2904	18.31	0.37	1.20	0.17
4C	na	na	na	na	na
5M	589	2.78	8.18	6.9	0.05
5F	2611	20.2	5.16	4.67	0.3
5C	na	na	na	na	na
					

Also included are two children (C).

### Long hair study

**Figure 1:**
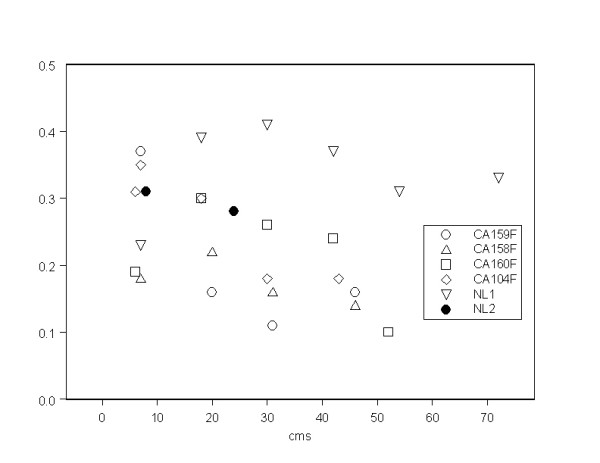
**Uranium (mg/kg, ppm) along the length of the hair locks of individuals in the long hair study (data from Table 4)**.

**Figure 2:**
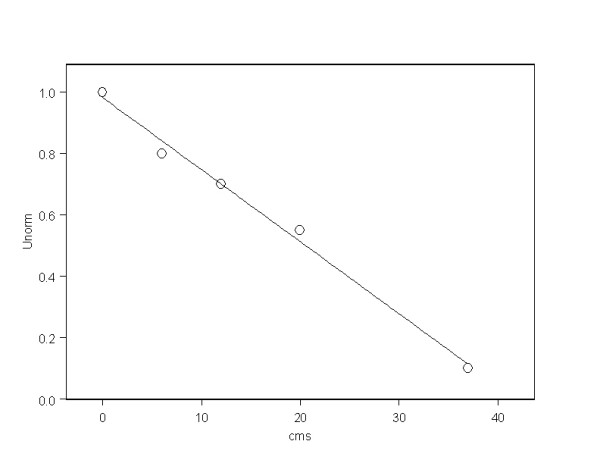
**Reduction of Uranium concentration along the hair lock for Swedish cases reported by Rodushkin and Axelsson**[46].

To further investigate the idea that historic exposure changes may be looked for along the length of hair, data for 4 women with long hair from the original group and two new women are given in Table 5. The results for Uranium in the scalp end of the lock for the women whose hair was taken in May 2010 and measured in the first set of tests agree well with the results obtained in the second set of measurements on hair taken 9 months later in February 2011. Figure 1 shows the variation in Uranium concentration along the length of the hair of the different individuals plotted against the mean period of the hair growth. In Figure 2 for comparison is the normalized concentration of Uranium along the length of locks of hair reported in a study of children living in Northern Sweden [42].

**Table 5:** Uranium levels ppm (mg/kg) in samples of hair taken in Feb 2011 along the length of the lock of women with CA children tested in the first study and two women volunteers N1 (with very long hair) and N2

*Cm from scalp*	*Date growing^a^*	*CA159F*	*CA158F*	*CA160F*	*CA104F*	*NL1*	*NL2*
First analysis	May 2010	0.16	0.23	0.24	0.31	NA	NA
6	Aug 2010			0.19	0.35	0.23	
7	July 2010	0.37	0.18				
8	June 2010						0.31
18	Aug 2009			0.30	0.30	0.39	
20	Jun 2009	0.16	0.22				
24	Feb 2009						0.28
30	Aug 2008			0.26	0.18	0.41	
31	July 2008	0.11	0.16				
42	Aug 2007			0.24		0.37	
43	July 2007				0.18		
46	Apr 2007	0.16	0.14				
52	Oct 2006			0.10			
54	Aug 2006					0.31	
72	Feb 2005					0.33	

^a ^assuming a growth of 1 cm a month

### Soil and water

Concentrations of Uranium in soil and water samples are given in Table 6. Soil samples show low concentrations with a mean of 0.76 ppm (9.4 Bqkg^-1^) SD 0.42 ppm and range 0.1-1.5 ppm; (N = 18). Levels in tap water were 2.28 μgL^-1^, water from a well was 2.72 μgL^-1 ^and from the river Euphrates as it flows though the town by the bridge 2.24 μgL^-1^.

**Table 6:** Literature data and this study on Uranium concentrations in hair of occupationally unexposed persons

*Publication*	*Country*	*N determinations*	*Mean mg/kg*	*Range mg/kg*
This study initial M + F May 2010	Iraq Fallujah	25 M + F	0.16	0.02-0.40
This study (long hair) F Feb 2011	Iraq Fallujah	29 female	0.256	0.10-0.41
Muikku et al [40]	Finland (high natural U)	852	0.216	0.0005-140
Akamine et al [60,61]	Brazil	22	0.0154	0.0021-0.0498
Rodushkin and Axelsson [43]	Sweden (high natural U)	114	0.057	0.006-0.436
Gonnen et al [45]	Israel	99	0.062	0.01-0.18
Byrne and Benedik [62]	Slovenia	17	0.0136	0.0027-0.033
Imahori et al. [63]	Japan	67 male 81 female	0.038 0.051	0.005-0.39 0.0082-1.28

## Discussion

### Elements found in excess in the hair

The results show that the parents of the children from Fallujah diagnosed with major congenital defects have significant excess concentrations of a range of elements. The following elements were present in concentrations that were more than two standard deviations from the mean levels in an uncontaminated Swedish population: Ca, Mg, Co. Fe, Mn, V, Zn, Sr, Al, Ba, Bi, Ga, Pb, Hg, Pd and U (for women only). Some of these were present in very high excess relative to both the laboratory control ranges and also the Swedish controls: Ca, Mg, Sr, Al, Bi and Hg. In examining the data we have chosen to focus on Calcium, Strontium, Bismuth, Mercury and Uranium, the first three since they are unusual and might be associated with earlier environmental exposures, and the latter as it is a known cause of damage to the developing foetus. Methyl Mercury has been associated with congenital neurological disease in Japan, but the types of congenital anomaly concern brain development and learning difficulty and do not match those found in Fallujah, which are dominated by heart and circulatory system and neural tube defects [44]. Matrix analysis for cross correlations between all these elements in the cohort do not reveal any significant relationships between them. However there is one interesting finding. The concentrations of these elements in the fathers are generally significantly lower than the concentration in the mothers. For the specific case of Uranium, the statistical boxplot is given in Figure 1 of the concentrations in mothers, fathers and the mixed samples of hair. The difference between mothers and fathers is significant at the p < 0.05 level.

The increased levels of contamination in the mothers relative to the fathers is generally quite marked for all of the excess contaminants examined, as can be seen in Table 4. This dependence of elemental concentration on sex was found for a wide range of elements by Rodusshkin and Axelsson [42,43] who found that women had roughly twice the levels of all the elements studied here than men and this included Uranium.

However, Gonnen et al [45] measured Uranium in a group of individuals living in southern Israel and found no significant difference in Uranium levels in hair between men and women. The relatively low levels of Uranium found in the two children we studied might be expected on the basis of measurements made on aborted fetuses which showed that they had 10% of the Uranium content of their mothers [44]

### Uranium in the hair: the initial study

In attempting to identify the cause of the cancer and congenital disease in Fallujah we cannot believe that the elements found in excess and listed above could, under normal conditions of exposure, be the cause of such a remarkable level of disease, since none of them, including Uranium, are present at levels which exceed the various environmental limits placed on contamination by government regulations in the USA or Europe. As far as Uranium is concerned, there are many parts of the world where Uranium levels in drinking water and human hair exceed those found by us in this study and levels of congenital disease and cancer in such areas are not markedly increased; as an example we may contrast the 38-fold excess leukaemia rates found in the questionnaire study [1] with the study of Auvinen et al of leukaemia in Finland in those drinking well water high levels of Uranium [46]. The mean level of Uranium water in the leukaemia cases in Finland was 7 μgL^-1 ^and that of the controls was 5 μgL^-1^. However, this approach begs a number of questions. The arguments relating to the health effects of Uranium from weapons use have been predicated on a different type of Uranium exposure which seems to be inhalation of nanoparticle ceramic oxides. Below about 1 micrometer diameter these will be translocated to the lymphatic system where part of it may remain for more than ten years [6,7]. The excretion into urine (or hair) from the bloodstream of Uranium derived from this source is likely to be very slow [6,7,38]. The ionisation damage close to such a particle is likely to significantly higher than would be calculated on the basis of the molecular concentration in tissue, and the local molecular concentration of the UO_2_^++ ^ion, the solubilised form of Uranium in the body, very high. A similar argument has been made by Zuchetti in relation to excess cancer and congenital anomalies found near the Quirra range in Sardinia where DU weapons are believed to be tested [12]. This would drive an equilibrium concentration of Uranium bound on the DNA which would locally be very high. In addition, it has been argued, and indeed established, that Uranium amplifies the natural background gamma radiation owing to its high atomic number, though there is a question about the level of local radiation dose enhancement this produces [29,47-50]. Furthermore the predicted photoelectron enhancements [29] from this molecular source have not been addressed or measured although theoretically it can be predicted that they may be significant [49,50].

In looking to see whether the levels of Uranium in the hair of the mothers and father of the children with congenital disease could indicate a cause of the health problems in Fallujah there are three questions that must be asked. First, do these levels seem to be higher than control populations living in uncontaminated areas of the world? Second, are these levels representative of an unchanging natural background exposure though drinking water containing natural Uranium which is a result of high natural levels of environmental Uranium? Third, are the Uranium isotope ratios indicative of a natural Uranium source? We will attempt to address all three of these questions in turn.

### Are the levels of Uranium in hair in Fallujah too high?

There are a number of literature sources for Uranium in hair listed in Table 7. If we omit the Finland data (which was from an area with high natural Uranium) the mean level of the other 5 studies is 0.04 ppm with a standard deviation of 0.023. The mean level in our initial study was 0.16 ppm and so this is almost five standard deviations from this mean and for a normally distributed population this would highly significant. The highest levels in Table 7 apart from the Finland sample are from an apparently uncontaminated control population studied by Gonnen et al. who measured Uranium in a population of 99 individuals living in Southern Israel, in the Negev Desert in 1999 [45]. The mean and median values were found to be 0.062 and 0.05 ppm respectively and results showed that those younger than age 45 had significantly lower values than those who were older. Comparing means in a non parametric distribution may not be correct way of comparing two groups. Because we have the distribution and the Israeli study distribution is given [45] we compared the Fallujah distribution results with those reported in the Israeli study. The histogram distributions for Fallujah and the Israeli cases younger than age 45 (which should be strictly comparable with the parents of the Fallujah children who were all younger than 45) are shown in Figure 4. Since these are clearly not normal distributions we employed the Mann-Whitney U-Wilcoxon non-parametric statistical test to determine whether the concentration of Uranium in the Fallujah parents were significantly higher than those in the Israeli control group. The results show a significant (2-tailed) excess in the Fallujah cohort p = 0.016.

**Table 7:** Uranium concentrations, beta gamma dose rate at 30 cm (μGyh^-1^) and activity ratios (where measured) in surface soil river sediment and water samples (μgL^-1^) from Fallujah, Iraq

*Sample*	*^a^Beta/gamma*	*^b^Uranium mgkg^-1^*	*^c^U238/U235*
Soil 1	150	0.857, 0.685, 1.033	129
Soil 2	120	0.164, 0.231, 0.252	132
Soil 3	160	0.688, 0.759, 0.637	129
Soil 4	190	0.725, 0.603, 0.867	130
Soil 5	220	0.119, 0.738, 0.907	118
Soil 6	130	1.44, 1.51, 1.51	129
BP Horizon Oil		0.070, 0.073, 0.073	138
Sediment R. Euphrates		1.05 mgkg^-1^	NA
Well water		2.72 μgL^-1^	NA
River water		2.24 μgL^-1^	NA
Tap water		2.28 μgL^-1^	NA

Three separate aliquots were taken from each soil sample and results are shown. Limit of Detection was 0.002 mgkg^-1^. As a check on the ion exchange method, the Uranium atom ratio from the deep oil from the BP Horizon Macondo oil spill was run with the Fallujah soil samples.

^a ^nGyh^-1^; ^b ^mean of three runs is tabulated for each of three aliquots; LOD, U-235 = 0.0007; ^c ^Natural ratio is 137.88; these measurements are derived from counts using the ion-exchange extracted Uranium solutions. 95% CI limits for Natural Uranium Ratio are 132.1 < Ratio < 144.1, Values below 132.1 are thus enriched, above 144.1 depleted with p < 0.05. NA = not assessed.

### Are the levels in hair a consequence of a locally high level of Uranium?

To examine this we measured Uranium in water samples from a well, from the river Euphrates and from tap water. The results in Table 6 show that there was 2.3 μg L^-1 ^total Uranium in the tap water and much the same level in both water from a local well and from the River Euphrates where it flowed through the town. It is generally accepted that the main source of Uranium in humans is from drinking water [45]. A compartmental biokinetic model of Uranium in human hair has been developed by Li et al. [51] and these authors show a correlation between Uranium intake in water and concentrations in hair for intakes greater than 10 μg per day. There is a very wide degree of scatter in the data at the low end with results falling between 0.2 and 1.0 ppm in the hair. However no data is given for intakes below 10 μg per day. Nevertheless since we have compared the Fallujah group with the Israeli group of Gonnen et al we may also note that these authors also measured Uranium in the drinking water. They reported that the Uranium in drinking water varied between 0.7 and 5 μg L^-1 ^[45]. It is therefore rather curious that at these levels the hair concentrations should be lower than the level in the Fallujah group but we cannot make too much of this since the standard deviations of the Uranium in drinking water in the Gonnen et al study were not given. Levels of total Uranium in the soil were measured and these were also not high in terms of natural background levels of Uranium in the world environment (Table 6). Levels in soil were generally less than 1 ppm (12 Bqkg^-1^) in Fallujah compared with average global soil levels of 1.8 ppm (22 Bqkg^-1^) [52]. Therefore the soil levels do not explain the total Uranium levels in the group. However there is a final question which is asked: is the Uranium natural?

### The Uranium isotope ratios

The use of DU weapons as anti-tank penetrators in Gulf War 1 led to attempts to track its use by means of measuring Uranium isotope ratios. One of us (CB) was involved with the UK Ministry of Defence in developing a urine test to study the levels of DU in veterans [38]. The rapid advances in technology which occurred in the late 1990s led to the development of ICPMS for detecting DU on the basis of its characteristic isotopic ratio signature and these machines became increasingly able to detect DU isotope signatures in urine tests. The natural atom ratio of U238/U235 is 137.88. Pure DU has a signature greater than 400 [6] but for the purposes of the urine test developed with the instruments in use at the time (2003) any ratio above 142 was considered to originate from a DU contamination [38]. If DU had been used in Fallujah, therefore, it might be expected that some deviation from the natural signature of 137.88 would be found if we looked. Measurements made on the soil samples quickly showed that although the total Uranium levels could be accurately determined, because of interference from other elements taken up in the acid dissolution of the sample, the concentrations of U235 needed to accurately define the isotope ratio were too low. Accordingly, an ion exchange extraction technique was developed, and results of these measurements showed clearly that the Uranium in the soil was not natural. It was not, however, depleted Uranium. It was, in fact, slightly enriched, with ratios varying from 118 to 132. Under the conditions of the extraction we are able to assess the 95% CI limits from the count variance found in relation to the total counts. We are able to say that for defining natural Uranium the Ratios must fall in the range 132.1 < Ratio < 144.1. Values below 132.1 are thus enriched, above 144.1 depleted with p < 0.05.

In the case of the hair samples for those samples with more than 0.1 ppm we were able to determine this ratio directly since the solutions had less interference and the instrument employed (octupole reaction cell) had a greater intrinsic sensitivity. At low Uranium levels, where overall U-235 concentrations become uncertain, it is technically possible to ignore overall accuracy in concentration and obtain isotopic ratios directly by dividing out the counts per second per channel. ICPMS counts atoms according to their mass, so the ratio of counts in the U238 channel to the U235 channel gives the isotope ratio without any assumptions about concentration. Ultimately this method fails at very low concentrations where the counts drop towards a zero concentration point of electronic amplifier background noise. This noise is a higher proportion of the U-235 channel counts than the U-238 channel counts since the latter are 137.88 times greater in natural Uranium. Thus any material will begin to appear enriched as the concentration falls towards the limit of detection. But as the concentration increases above this the measurements will converge towards the accurate value. Figure 5 shows the isotopic ratio U238/U235 plotted against total counts for U238. If we employ the data based on counts in the U235 channel of above 1000 counts the result (n = 14) is an isotope ratio of 135.16 with a standard deviation of 1.45. Since this is 2 standard deviations away from the natural Uranium value of 137.88, this results shows that the Uranium in the parents is slightly enriched. If the single outlier at 138.89 is removed the mean falls to 134.9 with a standard deviation of 1.02 which suggests that the outlier is just that. The SD is now almost 4 standard deviations away from the mean and the data is very tightly defined. This discovery of enriched Uranium in the hair might be expected since the soil sample data also shows traces of enriched Uranium. Note that the limit of detection for the instrument is conventionally placed at 1000 counts and that 4000 counts represents a concentration of approximately 0.003 ppm.

### Long hair study

**Figure 3:**
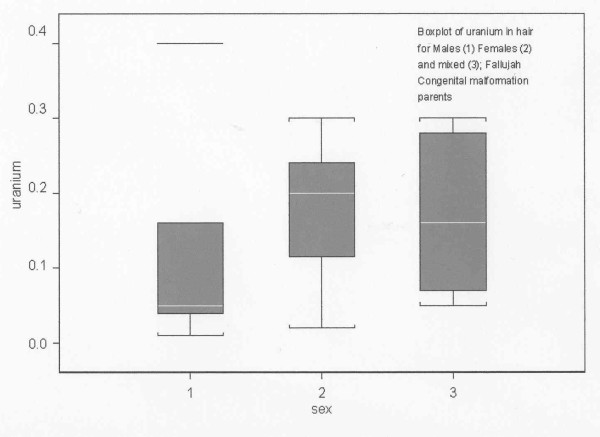
**Box plot of Ranges of Uranium concentrations in (1) Fathers (2) Mothers and (3) Mixed hair from both**.

If the higher levels of Uranium in the mothers relative to the fathers reflects increases in historic exposures then we may expect, under simplistic assumptions, to find an increasing trend in concentration in samples of hair taken from the proximal (scalp) to the distal end of the mothers. But the concentration of Uranium along locks of hair from the scalp to the distal end was investigated by Rodushkin and Axelsson [42,43]. These authors showed that for Uranium the concentration falls rapidly and regularly by distance from the scalp, presumably as a result of washing the hair. The trend was found to be such that 20 cm from the scalp the concentration was about half that at the scalp and at 37-39 cm from the scalp the concentration was about 15% of its initial value (Figure 2). In our study of the long locks of hair from 6 women from Fallujah we see clearly that the Uranium content does not fall along the hair as would be predicted by the findings of Rodushkin and Axelsson [42,43]. Figures 2 and 3 show this clearly. In the results of the 6 women we find individual variations in trends: four women have increasing levels of Uranium along the lock to the distal end, whilst two have falling levels. But even in the woman where the level falls most rapidly, the reduction over a period of 40 months (40 cm) is modest, perhaps to about 50% of the initial value. The results from Sweden suggest that the expected fall over this period should be 90% to a value about one tenth the original. Taken as a whole, including the women where the trend is upwards or level we have to conclude that the Uranium exposure 40 months before we took the samples was conservatively about five to ten times what is indicated now. Thus if we assume that (because of hair washing) the Uranium is lost from the women in Fallujah at the same rate as it is lost from the individuals in Sweden examined by Rodushkin and Axelsson [52] then 40 months before February 2011 (i.e. in Summer 2007) the levels would have been about 2.6 ppm rather than the 0.26 ppm measured in February 2011. And we looked for a woman with very long hair to investigate this historic exposure. This woman, NM1 has hair which is more than 80 cms long. It is clear that the trend in Uranium in her hair is fairly uniform, or maybe even rises. It is clear that the Uranium level in her hair at 40 cm indicates that if it had been measured in June 2007 it would have contained over 4 ppm of Uranium. We must assume that the loss of Uranium (through washing) is related to the number of washes, and so in this case, where the hair takes us back to 2005 (Table 4) the levels in the hair at that time must have been very high indeed.

**Figure 4:**
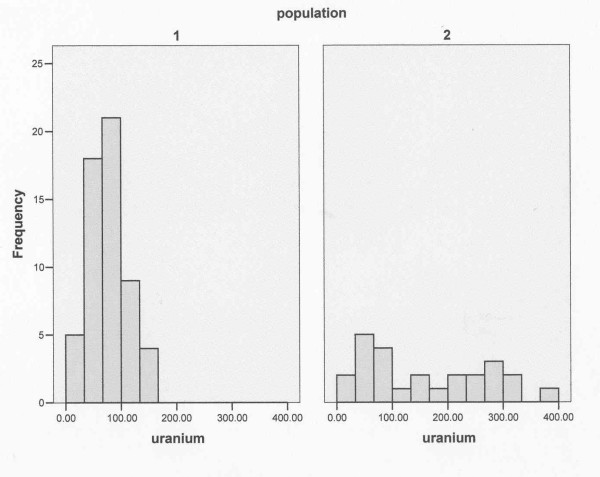
**Frequency histograms of Uranium concentration in two populations**. (1) Israeli control group (13) and (2) Fallujah congenital anomaly parents.

**Figure 5:**
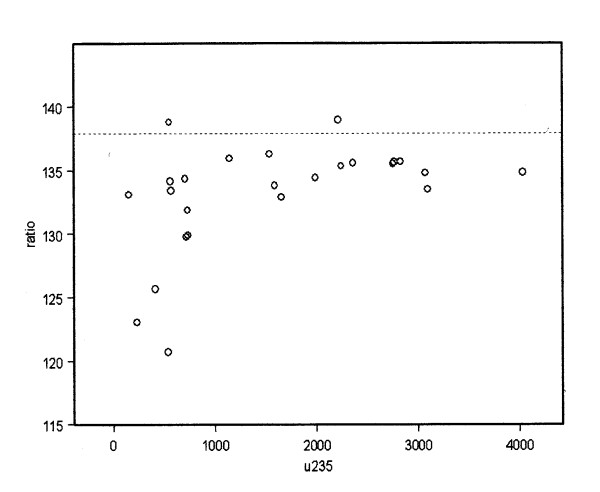
**Uranium isotope ratio plotted against the counts per channel for U235**. for all the parents in the study. Note effect of low counts and convergence of ratio R. for all points greater than 1000 cps towards a value of R = 135.16; SD 1.45 (n = 14). dotted line shows natural ratio of 137.9.

Thus we can provisionally conclude that the levels of Uranium in the hair of the parents of the congenital anomaly children are higher than could be easily explained by the environmental levels but more important the Uranium in the hair, the drinking water and in the environment has a component which is man-made and is enriched in the isotope U-235.

### Weaponised Uranium as a cause of the health effects in Fallujah

The use of passive DU munitions in Iraq (and also in the Balkans) has been admitted by the USA and UK forces and recent research, as outlined above, has suggested that Uranium may demonstrate anomalously high genotoxicity [53]. We have found high genotoxicity in this population: high cancer levels and high congenital anomaly levels. High childhood cancer levels in 1994-98 were also found in southern Iraq, where the main exposures to DU occurred in 1991. Significant excesses of congenital anomalies have been reported in the offspring of the Gulf veterans. However, DU has not been found. What we have found is slightly enriched Uranium (EU). We have found this in hair and in soil samples, using different instruments in different laboratories and employing different methods. We suggest that the levels in the hair seem to be high for the levels in the drinking water to be the sole source. In addition, we have found from the long hair study that the excretion into hair was much higher in the past than it is now. From the calculations made by the Royal Society [6,7] and the analyses of the UK DUOB [38] it would seem that the most likely explanation is that these excretions were the consequence of the slow dissolution and excretion of Uranium from some depot in the human body following an initial acute exposure. There remain two questions. Why use Uranium weapons in Fallujah? Why use enriched Uranium?

The military have been clear that although they used 350 tonnes in Gulf War 1, they did not employ Depleted Uranium weapons to any extent in Gulf War 2. That is *they did not use DU*. And indeed, no DU was found in the urine of GW2 veterans; one of us (CB) obtained the data from the 400 or so UK troops and although the urine Uranium levels were significantly high, the isotopic ratios showed them not to contain DU [38]. If anything, the distribution was rather broad and suggested that the ratios might contain some degree of enrichment. So one suggestion that may occur is that the use of EU or natural Uranium in weapons, although costly, enables the military to employ Uranium weapons and avoid subsequent detection [53]. They can truthfully say that they *did not use DU*.

But what of the second question? Uranium weapons are passive anti-armour weapons. Why would they be employed in house to house fighting against insurgents?

To examine this issue we turn to accounts of the Battle of Fallujah and the weapons used.

### The Battle of Fallujah

In 2004 Fallujah experienced two major military operations. The first siege and battle of Fallujah - *Operation Vigilant Resolve *- ran from 4^th ^April to 10^th ^May after the death of four US contractors. US forces attempted to control the city with ground operations and air support bombing but had to withdraw. The second siege and battle of Fallujah - *Operation Phantom Fury *- ran from 7^th ^November to 23^rd^December. US and Iraqi forces were involved in the most intense urban combat operations in Iraq since April 2003.

US Marines carried out ground operations with support from armoured vehicles, mortars, tanks and artillery. Air support was provided from Cobra and Apache helicopters, AV-8B Harriers, F-16 and F-18 fighter-bombers, AC130s and UAVs [54]. A wide range of guided weapons were used to destroy suspected insurgent targets before infantry troops entered strongly defended buildings.

CAS (Close Air Support) weapons included AGM-114 Hellfire, AGM-65 Maverick and TOW missiles. These mainly use shaped charge warheads believed to contain Uranium shaped charge liners (concept identified in US Patent 4441428 [55,56]. CAS operations in Fallujah also used 500lb GBU-12, 38 and possibly larger hard target guided bombs e.g. GBU-24 for hard targets and suspected bunkers. The advanced penetrator warhead versions of these (BLU-110, 111) use high-density metal ballast - either tungsten or Uranium [54,55,57]. Undepleted or low enriched Uranium contamination has been found in samples from heavily bombed locations in Afghanistan, Iraq and Lebanon [53,56,58].

US forces also used Fallujah to combat-test prototypes of at least two new types of thermobaric weapons - Thermobaric Hellfire missiles - AGM-114N [54]- and a new thermobaric RPG called SMAW-NE (Shoulder-fired, Multi-purpose Assault Weapon - Novel Explosive) [54]. Their effective performance in Fallujah led to major production contracts in 2005. Thermobaric weapons use high temperature/high pressure explosives as anti-personnel incendiary weapons. They char or vaporise victims in the immediate target location, or suffocate and collapse internal organs with their extended blast/vacuum effects [54]. These weapons use a new generation of reactive metal explosives, some of which are suspected of using Uranium for the high temperature and increased kinetic blast effects. If Uranium enhanced warheads were used in Fallujah these may have contained between 10 and 100 kgs of Uranium per warhead, depending on weapon type [56].

Finally, it is conceivable that these traces of enriched Uranium found in the present study relate to a completely new type of weapon or indeed to some other source entirely. But with regard to this we note that Fallujah is not the only war theatre where enriched Uranium has been detected. Measurements made on soil samples taken from a crater in Khiam in the Lebanon made by an Israeli missile found high levels of Uranium with significant enrichment ratios [53,56]. These results were obtained from two different laboratories using two different techniques, ICPMS and alpha spectroscopy. In addition, the crater was significantly radioactive shortly after the bomb fell but this radioactivity decayed away inside a few weeks. No fission products were found [59]. Enriched Uranium was also found in an ambulance air filter from the Lebanon [53,56,59]. We have also tested the Fallujah samples for radioactivity in the present study using long count time low resolution high sensitivity gamma spectroscopy and we have sent the water samples for measurements of Tritium. No man-made radionuclides were detected and no Tritium was found in the Fallujah samples.

The identity of the enriched Uranium weapons employed in Fallujah and elsewhere must remain an open question until the USA or Israeli military release more information.

## 5. Conclusions

This study analysed hair samples to examine contamination of the parents of children with congenital anomalies in Fallujah, a city where there was a very major and concentrated use of novel weapons in 2004. The purpose of our study was to identify the cause of the increased risks with some elemental component of the weaponry that was deployed. We conclude that the Fallujah parents of children with congenital malformations show unusually high levels of contamination by a number of chemical elements. These include Lead, Iron, Aluminium, Manganese, Strontium, Barium, Bismuth and Mercury. There are also high levels of contamination with elements that are derived from destroyed concrete and masonry, namely Calcium and Magnesium. However, none of these elements could account for the levels and types of ill health in the population, the cancer and congenital anomaly rates. In addition to these elements we found significant levels of Uranium, a material which has been associated with weapons employed in Iraq and in the Balkans since 1991 and also with genotoxicity. These levels were significantly higher than those expected on the basis of published control group data from various studies and particularly from Southern Israel. Further, the pattern of contamination with regard to hair length indicated a major contamination event in the past. The levels of Uranium could not be explained by any local Uranium deposits in the soil since measurements made of soil Uranium showed only modest concentrations though the Uranium was slightly enriched. Levels in the hair were also greater than could be easily explained by the levels we measured in the drinking water. The hair samples appear to contain traces of slightly enriched Uranium with an isotopic ratio of 135.16 (SD 1.5). The soil samples contained slightly enriched Uranium with isotope ratios ranging from 118 to 132 (natural range 95% CI 132.1-144.1) which demonstrates the existence of enriched Uranium in the Fallujah environment. Since none of the other elements found in excess in the parents were genotoxic except Uranium we conclude that these results support the belief that the effects in Fallujah follow the deployment of a Uranium-based weapon or weapons of some unknown type.

## 6. Authors Contributions

SA conceived of the study, diagnosed congenital anomalies, obtained volunteers and took hair samples.

MT diagnosed congenital anomalies, obtained volunteers and took hair samples.

CB assisted with the design of the study, analysed the data, drafted the manuscript and arranged for the analysis and interpretation of results of soil and water samples. MH assisted with the design of the study, organised the logistics, the collection of soil and water samples, the chain of custody and the transfer, division and recoding of the samples. EBB advised on analytical methodology and carried out the analytical measurements. All authors read and approved the final manuscript

## 7. Acknowledgements and conflict of interest

We are grateful to Dai Williams for useful discussions and information regarding weapons systems employed in Fallujah. One of us (CB) acknowledges core support from the Joseph Rowntree Charitable Trust during this work. We are grateful to the Cancer and Birth Defects Foundation, London, UK for assistance towards the cost of analytical measurements and the International Foundation for Research on Radioactivity Risk (Den Internationella Insamlingsstiftelsen för Forskning kring Radioaktivitetens Risker) Stockholm, Sweden, for some assistance towards equipment costs. None of us have any conflict of interest.
